# Retraction: Passive Immunization Reduces Behavioral and Neuropathological Deficits in an Alpha-Synuclein Transgenic Model of Lewy Body Disease

**DOI:** 10.1371/journal.pone.0313936

**Published:** 2024-11-13

**Authors:** The PLOS ONE Editors

Following the publication of this article [1], concerns were raised regarding results presented in Figs 3, 5, 9, S1, and S2. Specifically,

- There appear to be similarities between lanes within numerous panels presented in:
  ○ Fig 3G
  ○ Fig 5A, 5G
  ○ Fig 9A
  ○ Fig S2K
- There appear to irregularities suggestive of splice lines in the following panels:
  ○ Fig 5D Anti-FL-α-syn Insoluble panel
  ○ Fig 5J Anti-CC-α-syn Insoluble panel
  ○ Fig S1 9E4, Anti-FL-α-syn, and Anti-CC-α-syn panels
  ○ Fig S2K actin panel
- The following panels appear similar:
  ○ The Fig 5A actin panel and the Fig 5G actin panel
  ○ The Fig 5D actin panel and the Fig 5J actin panel

Regarding the similarities between and within the western blot panels, the corresponding author stated that the quality of the western blot panels presented in the article is not optimal and that close examination of these panels shows subtle differences between the lanes and bands that appear similar. Furthermore, the corresponding author commented that the vertical irregularities may be due to rearrangement of lanes to support illustration and regrets that splice lines were not clearly marked to indicate that these lanes represent individual, but not consecutive, lanes originating from the same blot. In addition, the corresponding author commented that the Fig 5A and 5G actin panels, and the Fig 5D and 5J actin panels intentionally represent the same results as these correspond to the same blots that were re-probed for full-synuclein, c-term nuclein, and actin. The corresponding author did not provide high resolution image data underlying the published results. PLOS remains concerned that the areas in the panels listed above appear more similar than would be expected from independent results, and in the absence of uncropped high resolution image data, these concerns cannot be resolved.

In light of the above concerns that question the reliability and integrity of the published results, the *PLOS ONE* Editors retract this article.

EM did not agree with the retraction. ER, MM, LC, BS, AA, CP, MT, KU, TTR, SMS, PS, RB, LM, MB, DG, and DS either did not respond directly or could not be reached.
